# A Rapid and Efficient Method to Obtain Photosynthetic Cell Suspension Cultures of *Arabidopsis thaliana*

**DOI:** 10.3389/fpls.2017.01444

**Published:** 2017-08-18

**Authors:** Simone Sello, Roberto Moscatiello, Nicoletta La Rocca, Barbara Baldan, Lorella Navazio

**Affiliations:** ^1^Department of Biology, University of Padova Padova, Italy; ^2^Botanical Garden, University of Padova Padova, Italy

**Keywords:** *Arabidopsis thaliana*, green calli, chloroplasts, photosynthetic cell suspension cultures, phytohormones

## Abstract

Photosynthetic cell suspension cultures are a useful experimental system to analyze a variety of physiological processes, bypassing the structural complexity of the plant organism *in toto*. Nevertheless, cell cultures containing functional chloroplasts are quite difficult to obtain, and this process is usually laborious and time-consuming. In this work a novel and rapid method to set up photosynthetic cell suspension cultures from the model plant *Arabidopsis thaliana* was developed. The direct germination of *Arabidopsis* seeds on a sucrose-containing agarized culture medium supplemented with 0.25 μg/ml 6-benzylaminopurine and 0.5 μg/ml 2,4-dichlorophenoxyacetic acid caused the straightforward formation of green calli at the level of seedling hypocotyls. The subsequent transfer of these calli in liquid culture medium containing the same concentrations of phytohormones and gradually decreasing sucrose levels allowed for the establishment of chloroplast-containing cell suspension cultures, containing functional chloroplasts, in a much faster way than previously described procedures. Pulse amplitude modulation analyses, measurements of oxygen evolution and electron transport rate, together with confocal and electron microscopy observations, confirmed the photosynthetic efficiency of these cell suspension cultures. The described procedure lends itself as a simple and effective way to obtain a convenient tool for a wide array of structural and functional studies on chloroplasts.

## Introduction

Plant cell suspension cultures are widely used as a useful and versatile experimental system to analyze complex plant physiological processes at the cellular and molecular level. By resolving the complexity of an *in toto* plant into its elementary units, suspension-cultured cells often represent a convenient tool to study a wide range of phenomena. This type of experimental system has been shown to be suitable to investigate many aspects of ion transport, secondary metabolite production, gene expression and defense responses (Ebel and Mithöfer, 1998; Hall, 2000; Moscatiello et al., 2006; González-Pérez et al., 2011; Wilson et al., 2014). In the field of signal transduction, cell suspension cultures have been shown to be an excellent mean to investigate calcium-mediated signaling events, by allowing the detection and amplification of even faint calcium signals, sometimes limited *in vivo* to only a particular tissue or cell type (Mithöfer et al., 1999; Lecourieux et al., 2005; Navazio et al., 2007a,b). Homogeneous plant cell populations were also used to evaluate the translocating properties of cell-penetrating peptides, i.e., short cationic peptides that can be used as nanocarriers for the intracellular delivery of proteins (Zonin et al., 2011). Special types of cell cultures, containing for example specific members of the plastid family, may help to decipher molecular, physiological and metabolic mechanisms more easily than an *in toto* system, based on the use of entire seedlings. Unfortunately, due to so far elusive reasons, photoautotrophic cell cultures are quite difficult to obtain, and have been developed only for a restrict number of species (Roitsch and Sinha, 2002). Previous studies have already shown the advantages of working with cell cultures containing functional chloroplasts (Roitsch and Sinha, 2002; Hampp et al., 2012; Gutiérrez et al., 2014). The use of both heterotrophic and autotrophic cell suspension cultures in signaling studies has recently allowed the dissection of differential stimulus-specific calcium signals of non-green plastids *versus* chloroplasts (Sello et al., 2016).

In this work we describe a novel and rapid method to obtain *Arabidopsis thaliana* photosynthetic cell suspension cultures, i.e., containing chloroplasts as functional type of plastids. The time interval needed to establish such experimental system was found to be greatly reduced in comparison with traditionally used methods to set up photoautotrophic cell cultures (Loyola-Vargas and Vázquez-Flota, 2006; Mustafa et al., 2011; Hampp et al., 2012; Sello et al., 2016).

## Materials and Methods

### Plant Material and Establishment of Photosynthetic Cultures

*Arabidopsis thaliana* ecotype Columbia (Col-0) seeds were surface sterilized for 60 s in a 70% ethanol, 0.05% Triton X-100 solution, 60 s in 100% ethanol and let dry on an autoclaved Whatman paper disk for at least 10 min. Twenty seeds were sown per cell culture dish (100 mm × 20 mm, Falcon, Corning, NY, United States), each containing 20 ml Murashige and Skoog (Murashige and Skoog, 1962) medium (MS Medium including vitamins, Prod. No. M0222, Duchefa Biochemie, Harlem, The Netherlands) supplemented with 3% sucrose, 0.25 μg/ml 6-benzylaminopurine (BAP), 0.5 μg/ml 2,4-dichlorophenoxyacetic acid (2,4-D), 0.8% plant agar, pH 5.5 and maintained in a growing chamber at 24°C with a 16/8 h light/dark cycle. After exactly 3 weeks, well-developed green calli, forming at the hypocotyl level, were cut and axenically transferred into 50 ml Erlenmeyer flasks (10 calli per flask) containing 10 ml MS medium supplemented with 2% sucrose, 0.25 μg/ml BAP, 0.5 μg/ml 2,4-D, pH 5.5 (pH 5.0 after autoclave). *In vitro* cultures were placed on a shaker at 80 rpm at 24°C under a relatively high illumination rate (110 μmol photons m^-2^ s^-1^) under an unvaried 16/8 h light/dark photoperiod and renewed every week by transferring 1 packed cell volume (PCV) in 20 ml of MS medium containing the same concentrations of phytohormones as above and stepwise 50% less sucrose content. The growth curve of the cell cultures was determined as previously described (Moscatiello et al., 2013).

### Light and Electron Microscopy Analyses

Observation of calli under a fluorescence stereomicroscope and of suspension-cultured cells by laser scanning confocal microscopy was carried out as previously described (Sello et al., 2016).

Transmission electron microscopy (TEM) analyses were carried as described by Zuppini et al. (2010).

### Determination of Photosynthetic Pigments

Total chlorophylls and carotenoids were extracted in 80% acetone (v/v). Suspension-cultured cells (2 ml) in exponential growth phase were pelletted by centrifugation (1600 rpm for 2 min), the culture medium was removed and the fresh weight annotated. 80% (v/v) acetone (1 ml) was added and the samples were incubated at 4°C for 48 h in the dark. The supernatant obtained by centrifugation was spectrophotometrically analyzed and chlorophyll a and b, and carotenoid concentrations were calculated according to Wellburn (1994) and referred to g of fresh weight and total protein content (Zonin et al., 2011).

### Pulse Amplitude Modulation (PAM) Analyses

Suspension-cultured cells (6-day-old) were placed in Petri dishes in a Closed FluorCam 800 MF (Photon Systems Instruments, Drasov, Czech Republic) and the maximum quantum yield of photosystem II (F_v_/F_m_, where F_v_ is the variable fluorescence given by the difference between the maximal (F_m_) and the basal (F_0_) fluorescence of chlorophyll) was analyzed and recorded.

### Measurement of Oxygen Evolution

The measurements were performed at 25°C by using a Clark-type O_2_ electrode (Hansatech, King’s Lynn, United Kingdom). Suspension-cultured cells (6-day-old) in exponential growth phase were harvested by centrifugation and suspended in fresh culture medium. Their respiratory rate was measured in the dark while O_2_ evolution was recorded after application of light with an intensity of 1500 μmol photons m^-2^ s^-1^ and in presence of 5 mM bicarbonate. Measurements were carried out in 2 ml total volume of cell culture. The total O_2_ production was referred to g of cell fresh weight.

### Measurement of Electron Transport Rate

Electron transport rate (ETR) of dark-adapted green cell suspension cultures was characterized monitoring PSII chlorophyll fluorescence at different light intensities. After 20 min of dark adaptation to completely oxidize the photosynthetic electron transport chain, samples were treated with actinic light of increasing intensity. After 60 s of each treatment a saturation pulse of 6000 μmol photons m^-2^ s^-1^ (600 ms) was used to assess the redox state of PSII. Every analysis lasted 15 min in total. During this protocol chlorophyll fluorescence was monitored using a Dual-PAM 100 (Walz). ETR was calculated as Y(II) × PPFD × 0.5 (Maxwell and Johnson, 2000).

### Starch Detection and Quantitative Determination

Starch was detected by light microscopy observations after Lugol staining of photosynthetic cell suspension cultures and by TEM analyses. For starch quantitative determination, cells (>1 g fresh weight) were extensively washed with PBS (five times with 10 ml, each time followed by centrifugation at 450 *g* for 1 min). The cell pellet was ground in liquid nitrogen and starch concentration was measured by using a Starch Assay Kit (Sigma–Aldrich, St. Louis, MO, United States) according to manufacturer’s instructions.

## Results

**FIGURE 1:**
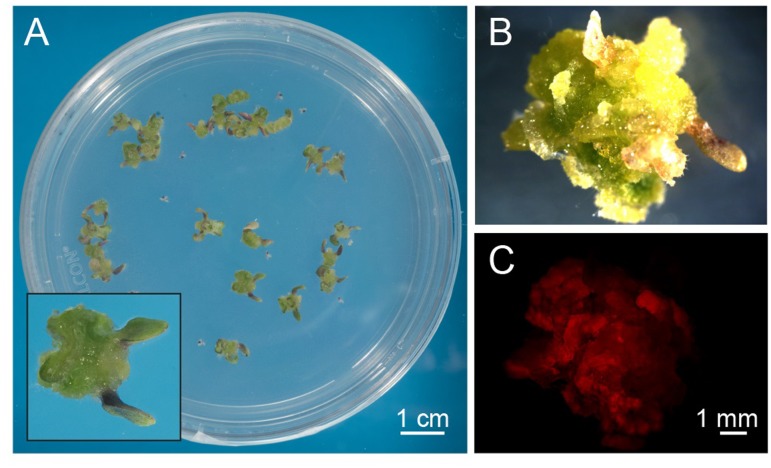
Exogenous hormone-induced production of green calli from *Arabidopsis* seedlings. **(A)** Surface-sterilized seeds were plated on a cytokynin- and auxin-enriched agarized solid medium containing sucrose (MS, 0.8% agar, 3% sucrose, 0.25 μg/ml BAP, 0.5 μg/ml 2,4-D) that had a delaying effect on seedling growth and development. Insert: magnification of a well-developed callus originating from the dedifferentiation and proliferation of hypocotyl cells after 3 weeks of germination. **(B,C)** Observations at the fluorescence stereomicroscope of green calli **(B)**, displaying red autofluorescence of chlorophyll when excited with blue light **(C)**.

**FIGURE 2:**
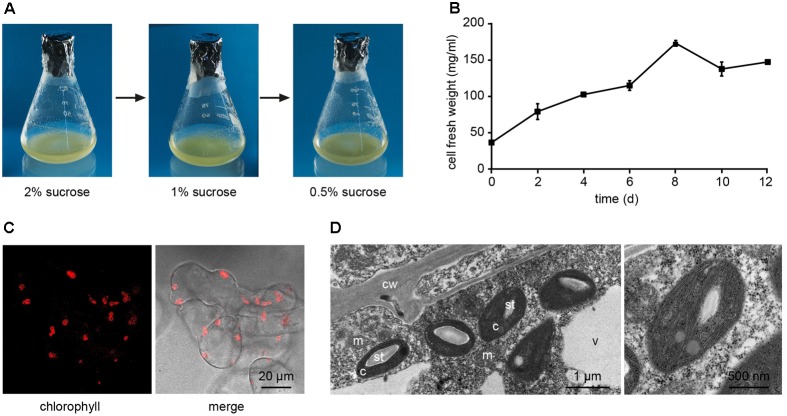
Establishment of *Arabidopsis* photosynthetic cell suspension cultures. **(A)** Suspension-cultured cells were maintained in MS culture medium containing 0.25 μg/ml BAP and 0.5 μg/ml 2,4-D, and gradually reduced sucrose concentrations to stimulate the photosynthetic activity. **(B)** Growth curve of the photosynthetic cell suspension culture, after three sub-culturing steps in 0.5% sucrose-containing medium. Data are the means ± SE of three independent replicates for each time point. **(C)** Confocal microscopy analysis confirmed the presence of chloroplasts as functional type of plastids in the cell suspension cultures. **(D)** Ultrastructure of chloroplast-containing suspension-cultured cells. c, chloroplasts; cw, cell wall; m, mitochondria; st, starch; v, vacuole. In the image on the right the magnification of a chloroplast is shown.

*Arabidopsis thaliana* seeds were surface sterilized and sown on Murashige and Skoog (MS) medium containing 3% sucrose, 0.8% plant agar and supplemented with 0.25 μg/ml 6-benzylaminopurine (BAP) and 0.5 μg/ml 2,4-dichlorophenoxyacetic acid (2,4-D). We observed that, already after 1 week from sowing, the growth of *Arabidopsis* seedlings was slowed down, with the subsequent progressive formation of a green mass of dedifferentiated cells at the hypocotyl level (**Figure 1A**). After 3 weeks well-developed green calli, exhibiting evident chlorophyll autofluorescence (**Figures 1B,C**), were axenically separated from the root and cotyledons, cut in small pieces with a sterilized sharp blade, and transferred into liquid MS medium containing 2% sucrose and the same concentrations of phytohormones described above. In particular, cell suspension cultures were initiated by transferring 10 green calli (0.14 ± 0.01 g fresh weight, *n* = 12) into 50 ml Erlenmeyer flasks containing 10 ml of liquid medium. Cell suspension cultures were renewed every week by transferring 1 PCV in 20 ml of MS medium containing stepwise 50% less sucrose content, in order to stimulate photosynthetic activity. In particular, sucrose concentration was gradually reduced from 2 to 1% and then to 0.5% (w/v) every 2–3 weeks (**Figure 2A**). Once stabilized in the lowest sucrose concentration condition, a growth curve for the newly established photosynthetic culture cell line was determined. The fresh weight of the cell cultures was found to increase about five times in 8 days, followed by a stationary phase lasting 4 more days (**Figure 2B**).

**FIGURE 3:**
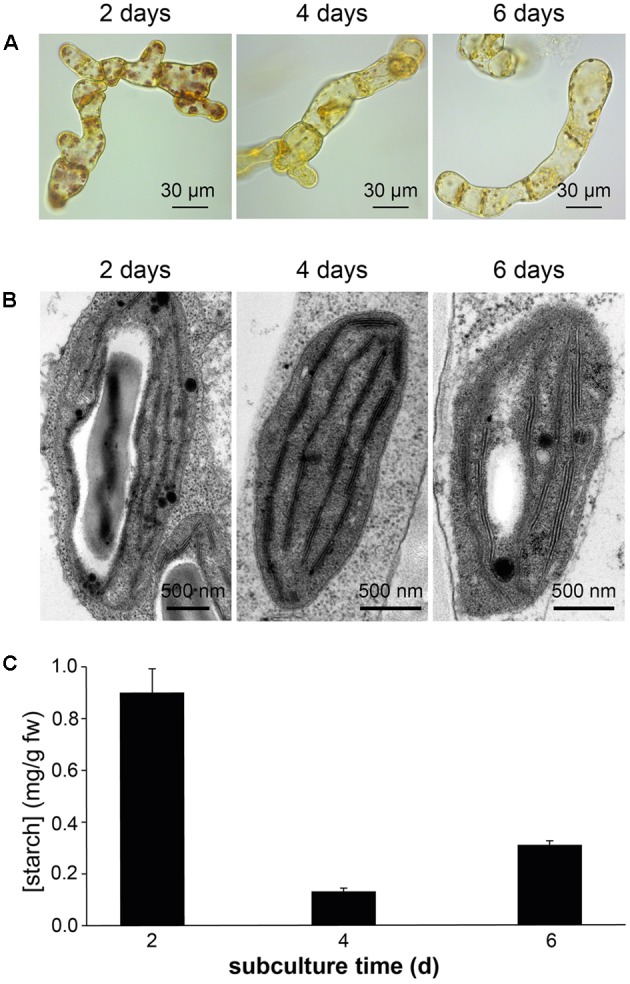
Detection of starch in *Arabidopsis* photosynthetic cell cultures at different subculture times. Determinations of starch was carried out in suspension-cultured cells after 2, 4, and 6 days of subculture by Lugol staining **(A)**, TEM analyses **(B),** and a quantitative biochemical assay **(C)**.

**FIGURE 4:**
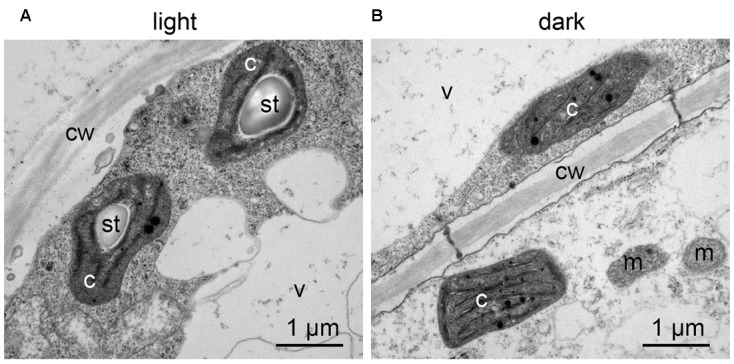
Transitory starch accumulation in *Arabidopsis* photosynthetic cell cultures during the light/dark cycle. TEM observations were carried out: **(A)** at the end of the 16 h light phase; **(B)** at the end of the 8 h dark phase. c, chloroplasts; cw, cell wall; m, mitochondria; st, starch; v, vacuole.

**FIGURE 5:**
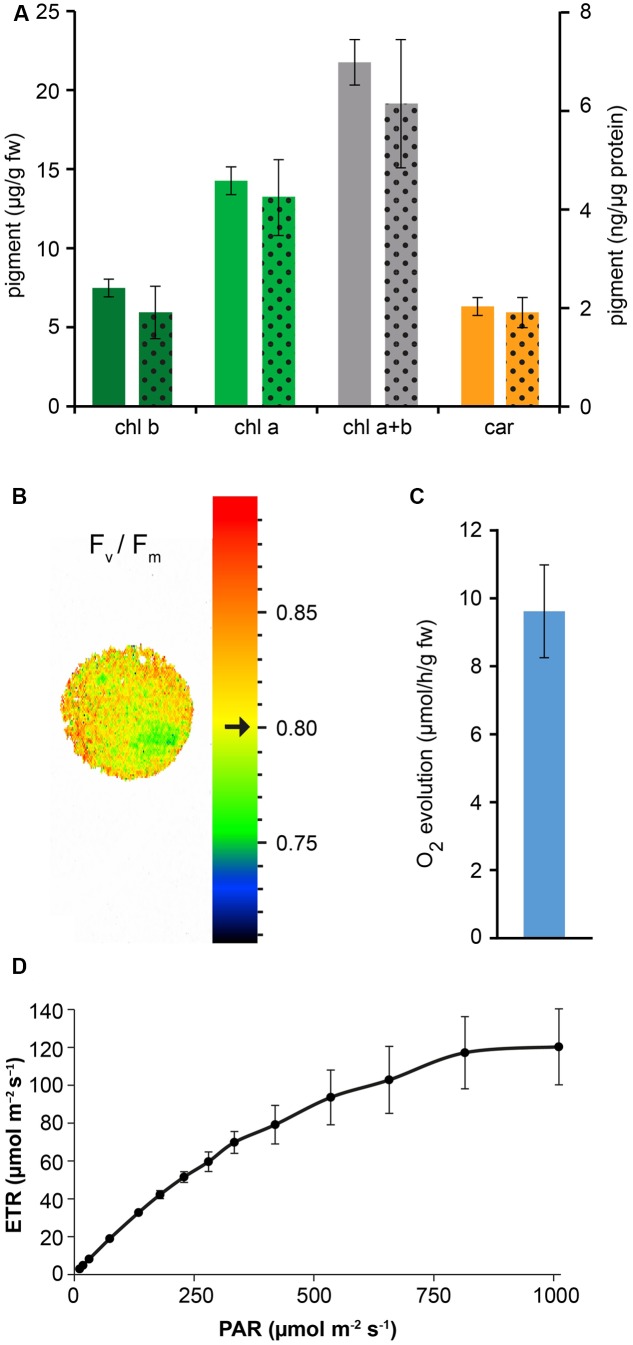
Measurement of photosynthetic pigment content and photosynthetic activity of *Arabidopsis* cell suspension cultures. **(A)** Quantitative analysis of chlorophyll b (chl b), chlorophyll a (chl a) and total carotenoids (car). Full column values refer to the y axis on the left, dotted column values refer to the y axis on the right. Data are the means ± SE (*n* = 5). **(B)** PAM imaging analysis demonstrated a very good maximum quantum yield of photosystem II. Black arrow indicates a F_v_/F_m_ mean value of 0.80 ± 0.01 (*n* = 3), representative for the displayed sample spot. **(C)** Quantitative analysis of O_2_ evolution. Data are the means ± SE (*n* = 8). **(D)** Electron transport rate (ETR) at PSII. Data are the means ± SE (*n* = 3).

Confocal microscopy (**Figure 2C**) and TEM (**Figure 2D**) analyses of suspension-cultured log-phase cells demonstrated the presence of chloroplasts as functional type of plastids in the newly established green cultures. In particular, TEM analyses confirmed the good ultrastructural organization of the cells, with chloroplasts exhibiting grana, stroma lamellae and starch granules (**Figure 2D**). Light microscopy observations after Lugol staining (**Figure 3A**), TEM analyses (**Figure 3B**) and biochemical analyses based on a quantitative starch assay (**Figure 3C**) demonstrated that the relative abundance of starch changed with the age of the culture, being maximal after 2 days from subculturing, minimal after 4 days, and reaching an intermediate level after 6 days. Transitory starch, produced during the 16 h light period, was found to disappear after 8 h of dark (**Figure 4**). Although the total photosynthetic pigment content of the cell suspension cultures (**Figure 5A**) was found to be at slightly lower levels than in a previously published work in *Arabidopsis* (Hampp et al., 2012), pulse amplitude modulation (PAM) analyses (**Figure 5B**) and measurements of the rate of oxygen evolution (**Figure 5C**) demonstrated the good photosynthetic efficiency of our experimental system. Moreover, photosynthetic analyses were carried out on cell suspension cultures also with the aim to assess the ETR of PSII at different light intensities. This parameter gives an indication on PSII efficiency in using light, therefore demonstrating PSII functionality. **Figure 5D** shows that ETR gradually increases from 0 to nearly 120 μmol e^-^ m^-2^ s^-1^ at 811 μmol photons m^-2^ s^-1^, following light intensity increase. Once reached this level, ETR is maintained at this steady-state also at 1000 μmol photons m^-2^ s^-1^. These ETR values are similar to those reported in literature obtained from *Arabidopsis* leaves, indicating that PSII in our photosynthetic cell suspension cultures are functionally comparable with the photosynthetic apparatus in entire leaves (Munekage et al., 2008; Sperdouli and Moustakas, 2012).

When suspension-cultured cells were transferred from 0.5% sucrose-containing medium to sucrose-free medium, although exhibiting a decreased growth rate, they were still found to be characterized by a good F_v_/F_m_ ratio (0.76 ± 0.01 at 0% sucrose *versus* 0.80 ± 0.01 at 0.5% sucrose; *n* = 3).

**FIGURE 6:**
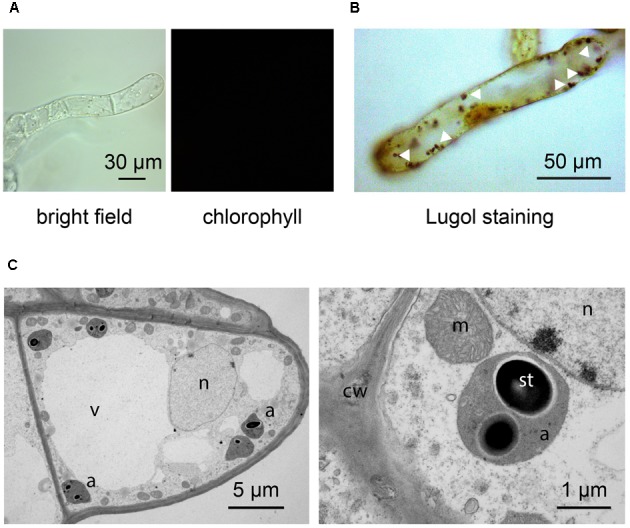
Set up of *Arabidopsis* heterotrophic cell suspension culture, starting from the previously established photosynthetic culture. **(A,B)** Light microscopy observations of suspension-cultured cells, demonstrating the lack of any chlorophyll fluorescence signal **(A)**, as well as the presence of evident Lugol-stained starch granules (white arrowheads) **(B)**. **(C)** Ultrastructure of amyloplast-containing suspension-cultured cells. a, amyloplasts; cw, cell wall; m, mitochondria; n, nucleus; st, starch; v, vacuole.

By applying our novel protocol, we were also able to set up heterotrophic cell suspension cultures directly from photoautotrophic ones, thus significantly shortening the required time interval. After obtaining photosynthetic cell suspension cultures, they were subcultured into MS medium, containing 0.25 μg/ml BAP 0.5 μg/ml 2,4-D, and supplemented with a high concentration of organic carbon (3% sucrose). Moreover, flasks were permanently wrapped in aluminum foil. After about 3 weeks, cells completely lost their ability to photosynthesize and plastids effectively turned from chloroplasts to amyloplasts, allowing for the obtainment of functional heterotrophic cell suspension cultures. The lack of chlorophyll autofluorescence (**Figure 6A**), together with the presence of evident Lugol-stained starch granules (**Figure 6B**) inside these organelles, whose ultrastructure appeared well-preserved (**Figure 6C**), confirmed the identity of these plastids as amyloplasts.

## Discussion

In this work a novel procedure to rapidly obtain photosynthetic *Arabidopsis* cell suspension cultures containing chloroplasts as functional type of plastids is presented. The novelty of this method relies on the initial germination of *Arabidopsis* seeds on a phytohormone-enriched medium, rather than on classical hormone-free media. The abnormal growth of *Arabidopsis* seedlings thus obtained, which is likely due to the crosstalk among endogenous and exogenous hormones, led to the dedifferentiation of cells and the formation of a well-evident green callus at the hypocotyl level after 3 weeks. The concentrations of phytohormones used (0.25 μg/ml BAP and 0.5 μg/ml 2,4-D) correspond to those traditionally employed to grow and maintain *Arabidopsis* heterotrophic cell suspension cultures (Moscatiello et al., 2013; Sello et al., 2016). In comparison with previously published methods (Mustafa et al., 2011; Hampp et al., 2012; Sello et al., 2016), the time frame needed to set up photoautotrophic cultures was found to be highly reduced (about 2–3 months *versus* 2 years). In a previously described more elaborate procedure, two-tiered flasks with a carbonate buffer in the lower compartment were used to maintain about 2% CO_2_ in the atmosphere (Hampp et al., 2012). In our experimental system, that more easily meets the standard basic equipment of every plant cell biology laboratory, cell suspension cultures were simply maintained in sterile Erlenmeyer flasks containing one-fifth volume of culture medium to ensure a proper aeration of the cell culture. Therefore, it is plausible that the comparatively lower concentration of pigments in our *in vitro* culture system may be due to a reduced rate of inorganic carbon availability. In agreement with this, we found that the presence of 0.5% sucrose in the medium was advisable to ensure a convenient cell growth rate (with a 7 days-subculture time), suitable to the performance of weekly experiments. Nevertheless, deprivation of sugar in the medium (0% sucrose) was not found to significantly alter the photosynthetic efficiency of the cell culture.

A diverse array of experiments can be conveniently performed in photosynthetic suspension-cultured cells, containing chloroplasts as unique type of plastids. For example, photoautotrophic cell cultures stably expressing the bioluminescent calcium reporter aequorin targeted to the chloroplast stroma were found to be an ideal experimental system to determine the specificity of organellar Ca^2+^ signaling (Sello et al., 2016).

Cell suspension cultures can also be useful as a valid alternative to *Arabidopsis* leaves as plant material source for the isolation of protoplasts. Indeed, protoplast isolation from cell suspension cultures is a quick, highly reproducible and straightforward procedure (Moscatiello et al., 2013) that typically gives high yields (from 10^5^ to 10^6^ protoplasts/ml of exponentially growing suspension-cultured cells). Similarly, intact chloroplasts can be directly isolated from cell suspension cultures, i.e., homogeneous cell populations that can be available every week in bulk quantities and with very reproducible conditions. Moreover, photosynthetic suspension-cultured cells and protoplasts are a convenient system to verify the localization of fluorescently tagged recombinant proteins targeted to different chloroplast sub-compartments (Sello et al., 2016; Navazio L., unpublished results).

The new protocol herewith described was found to be useful not only to rapidly obtain photoautotrophic cell suspension cultures, but also heterotrophic ones. Indeed, classical methods to set up *in vitro* cell cultures traditionally rely on the application of callus induction media to make hypocotyls and cotyledons dedifferentiate and generate calli. The complete procedure necessary to obtain rapidly proliferating cell suspension cultures containing non-green plastids involves a time-consuming procedure consisting of many different *in vitro* steps (Loyola-Vargas and Vázquez-Flota, 2006; Moscatiello et al., 2013; Barkla et al., 2014). An additional advantage of this method is that, by simply transferring the photosynthetic cultures from a 16 h light/8 h dark photoperiod to a condition of total darkness, in a culture medium containing a high concentration of sucrose (3%) as source of organic carbon, heterotrophic cell suspension cultures, containing amyloplasts rather than chloroplasts, can be easily and rapidly obtained, and maintained indefinitely *in vitro*.

## Conclusion

The procedure described in this work may represent a useful and straightforward tool to quickly obtain *Arabidopsis* cell suspension cultures containing functional chloroplasts, i.e., a convenient experimental system for a wide array of studies aimed at the analysis of chloroplast structure and function.

## Author Contributions

LN conceived and designed the work. SS, RM, NLR, BB, and LN performed the experiments and analyzed the data. SS and LN wrote the paper. All authors participated in editing the manuscript and approved its final version.

## Conflict of Interest Statement

The authors declare that the research was conducted in the absence of any commercial or financial relationships that could be construed as a potential conflict of interest.
